# Synergistic Potential of Argentatins A and B to Improve 5‐Fluorouracil Cytotoxicity in Colorectal Cancer Cell Models

**DOI:** 10.1111/jcmm.70294

**Published:** 2024-12-20

**Authors:** Paula Sánchez‐Olivares, Aniela M. Silva‐Nolasco, Miguel A. de la Cruz‐Morcillo, María Mercedes García‐Martínez, Alejandro Pinedo‐Serrano, Manuel Carmona, Eva M. Galán‐Moya

**Affiliations:** ^1^ Cancer Pathophysiology and Therapy Lab Institute of Biomedicine (IB‐UCLM) Universidad de Castilla‐La Mancha Albacete Spain; ^2^ Institute for Regional Development (IDR) Universidad de Castilla‐La Mancha Albacete Spain; ^3^ Universidad de Castilla‐La Mancha, E.T.S.I. Agronómica, de Montes y Biotecnología (ETSIAMB) Albacete Spain; ^4^ Instituto Técnico Agronómico Provincial de Albacete, ITAP Albacete Spain; ^5^ Facultad de Enfermería Universidad de Castilla‐La Mancha Albacete Spain

**Keywords:** 5‐fluorouracil, argentatins, cancer therapy, colorectal cancer, synergy

## Abstract

Colorectal cancer is the third most commonly diagnosed cancer worldwide and the second most common cause of cancer‐related death in both men and women. Although a number of treatments are available to combat this malignancy, the antimetabolite 5‐fluorouracil has been the cornerstone of therapy since its synthesis in the 1950s. Unfortunately, the prolonged use of 5‐fluorouracil can lead to chemoresistance, which has prompted research into combination regimens to improve efficacy and quality of life and reduce resistance. Here, we evaluated the synergistic potential of two compounds isolated from guayule, and argentatins A and B, alone and in combination with 5‐fluorouracil in a panel of colorectal cancer cell lines. Cell viability assays showed that the combination treatment (argentatin A with 5 fluorouracil) significantly enhanced cytotoxicity, especially in RKO, where the analysis using the Bliss independence model indicated a remarkable synergistic effect with the lowest doses of both compounds. In contrast to the combination with argentatin B, in which the additive effect was only found in the HCT‐116 cell line. Finally, immunocytometric analysis revealed that combination treatments induced higher rates of apoptosis than single‐agent treatments. Collectively, our findings indicate that argentatins A and B may enhance the anti‐tumour effects of 5‐fluorouracil and may represent a promising strategy to improve the efficacy of anticancer therapies based on this antimetabolite.

## 1 Introduction

Colorectal cancer (CRC) is the third most common malignancy and the second most deadly, with an estimated 1.9 million new cases and 0.9 million deaths worldwide in 2022 [1]. The incidence rate of CRC is approximately 14–20 per 100,000 people and is slightly higher in men than that in women [2]. The risk of developing CRC is low before the fifth decade of life, unless it is a hereditary form of the disease, but increases markedly thereafter.

Both genetic and environmental factors have been implicated in the aetiology of CRC, including family history (genetic mutations), poor dietary habits, microbiota dysbiosis and various inflammatory processes [3]. Treatment of CRC is largely based on surgical resection and adjuvant chemotherapy, but also includes neoadjuvant radiotherapy and targeted therapies. Unfortunately, drug resistance remains the major obstacle to the successful treatment of CRC. A classic example of drug resistance in CRC is the loss of activity of the chemotherapeutic antimetabolite 5‐fluorouracil. The cytotoxic effect of 5‐fluorouracil is mainly due to the inhibition of thymidylate synthase, the rate‐limiting enzyme for pyrimidine synthesis. Normally, thymidine binds to the pyrimidine base thymine, and 5‐fluorouracil (a fluorinated synthetic analog of pyrimidine) substitutes for thymine and inhibits DNA replication [4, 5, 6]. Cancer cells use several mechanisms to escape the cytotoxicity of 5‐fluorouracil, including increased DNA repair and drug metabolism.

New therapies are urgently needed to prevent such resistance and improve the management of patients with CRC. In this regard, natural products have been used for millennia in the treatment of many diseases, including cancer, and many new plant compounds are being tested in combination therapy to enhance the efficacy of classical chemotherapy [7]. For example, ursolic acid is a well‐known triterpenoid with proven antitumor activity and is found in more than 30 botanical species [8]. Ursolic acid has shown effects on cell viability and autophagy in breast cancer cells at concentrations as low as 20 μM, where it is able to block AKT activation, thereby promoting apoptosis and autophagy [9].

There is also increasing interest in the active secondary metabolites of *Parthenium argentatum* A. Grey (guayule) [10, 11]. Some of these metabolites with a triterpene structure, commonly known as argentatins [12], have previously been shown to have immunomodulatory [13], anti‐inflammatory [14, 15, 16] and analgesic [17] activities, through mechanisms that remain largely unknown [16]. Interestingly, argentatins also show antitumor activity in several cell lines derived from different types of cancer [18, 19, 20], and have been reported to reduce tumour growth in murine cancer models with less toxicity than cisplatin [18, 21]. The activity of the argentatins against various colon cancer cell lines has been reported to range from 24 to 61 μM [19, 21].

Drug combinations are a proven approach in complex diseases such as cancer, where the main objectives are to achieve a synergistic therapeutic effect at lower doses to reduce cytotoxicity, and to minimise or at least delay chemoresistance phenomena [22]. The aim of the present study was to evaluate the antitumor properties of argentatins (A and B) in combination with 5‐fluorouracil, with the ultimate goal of improving current therapies by combination with these natural compounds.

## 2 Experimental Procedures

### 2.1 Cell Lines

The CRC cell lines HCT‐116, HT‐29, RKO, SW‐620 and SW‐480 were cultured in Dulbecco's Modified Eagle's Medium (Gibco‐Invitrogen, Madrid, Spain) supplemented with inactivated 10% fetal bovine serum and 100 U/mL penicillin/streptomycin (all from PAN‐Biotech, Aidenbach, Germany). Cells were maintained under controlled conditions of temperature (37°C), atmospheric CO_2_ (5%) and relative humidity (95%). The culture medium was changed every 2–3 days and cells were passaged every 3–4 days using trypsin.

### 2.2 Isolation and Chemical Preparation of Argentatins

Two grams of guayule resin from AZ‐2 accession were dissolved in 3 mL of hexane and injected into a flash chromatography system (BÜCHI Pump Manager C‐615; Labortechnik, Flawil, Switzerland) with a reversed‐phase column (Biotage Sfär Silica D—Duo 60 μm; Biotage AB, Uppsala, Sweden), protected with a pre‐column (Biotage Sfär Silica Samplet 60 μm), at a flow rate of 60 mL min^−1^. Chromatographic separation was obtained by elution with organic solvents of increasing polarity (from hexane to ethyl acetate). Collected fractions of 280 mL containing the target compounds (confirmed by thin layer chromatography) were combined and concentrated in a rotary evaporator (Laborota 4000, Heidolph, Germany) at 210 mbar and 50°C. The purification yield was determined gravimetrically from the injected resin, 6.98% for argentatin A and 6.55% for argentatin B. Each isolated compound and its fractions were stored in the dark at 4°C until use. The isolated compounds were analysed by HPLC coupled to a refractive index detector and by LC‐mass spectrometry, as described [23, 24], and were compared against standards, kindly provided by Dr. Mariano Martínez‐Vázquez (Universidad Nacional Autónoma de México).

Argentatins A and B were dissolved in dimethylsulfoxide (DMSO; Sigma‐Aldrich, Madrid, Spain) at 20 mM, as previously described [19], and subsequently diluted in the cell culture medium at the indicated concentrations. A 5 mM stock of 5‐fluorouracil (F‐6627; Sigma‐Aldrich, Madrid, Spain) was also prepared in DMSO. All compounds were stored at −20°C after dilution.

### 2.3 Viability Assays

Sub‐confluent monolayer cultures were plated in 96‐well plates (5000 cells per well) and incubated for 24 h at 37°C and 5% CO_2_. After this, cells were treated with different concentrations of the drugs alone or in combination. Cell proliferation was then analysed after 72 h using an MTT‐based colorimetric assay. MTT (5 mg mL^−1^) was added to each well and plates were returned to the incubator for 1 h. The medium (MTT) was then removed and 100 μL of DMSO was added to each well, followed by agitation for 5 min in the dark to dissolve the MTT‐formazan crystals. The absorbance of the samples was then recorded at OD 570 nm, and the OD at 690 nm was used as a reference (SpectroStar Nano Reader, BMG Labtech, Ortenberg, Germany).

### 2.4 Combination Assays

To evaluate the combined effect of the argentatins and 5‐fluorouracil, different doses of argentatins A and B (IC_50_ and the IC_50_ reduced to 75% and 50%) were combined with 5‐fluorouracil in the same concentration range as the argentatins. The Bliss Independence model was used to analyse the drug combination data and characterise the type of pharmacological interaction. Results were analysed using the free software Combenefit (version 2.021), which analyses drug interactions between a specific pair of drugs and calculates the extent of the combined effect in terms of synergy and/or antagonism.

### 2.5 Generation of a Resistant Cell Model

The HCT‐116 cell line was initially treated with 1 μM of 5‐fluorouracil and, as a control, a plate of HCT‐116 was cultured in parallel in absence of treatment. Cells exposed to 5‐fluorpuracil were subdivided as they reached confluence, and the concentration of treatment was gradually increased, at regular time intervals, to allow for cell adaptation.

In parallel, dose–response assays were performed to assess cell viability and confirm the acquisition of resistance. After approximately 6 months of continuous and stepwise exposure to treatment, a resistant cell line capable of stable growth in the presence of 10 μM 5‐fluorouracil was established.

### 2.6 Flow Cytometry Assays

#### 2.6.1 Cell Cycle Analysis

The RKO cell line was seeded in 10‐mm^2^ plates at 500,000 cells/plate and incubated for 24 h at 37°C and 5% CO_2_. After this time, each plate of cells was treated with the dose corresponding to the IC_50_ of each compound obtained in the cell viability assays, and plates were incubated for 24 h as before. Cells were then collected with 750 μL of trypsin and fixed with 300 μL of ice‐cold 70% ethanol. The cell pellet was washed with 700 μL of PBS plus 2% bovine serum albumin and then incubated with 300 μL of propidium iodide/RNase staining solution (ImmunoStep SL, Salamanca, Spain) for 1 h at 4°C in the dark with agitation.

#### 2.6.2 Cell Death Analysis

The RKO cell line was seeded in 10‐mm^2^ plates at 500,000 cells/plate and incubated for 24 h at 37°C and 5% CO_2_. Each plate of cells was then treated with the dose corresponding to the IC_50_ of each compound obtained in the cell viability assays, and the plates were incubated for 72 h as before. Following this, adherent and floating cells were collected and stained with Annexin V Binding Buffer (Annexin V‐DT‐634 and propidium iodide; ImmunoStep) for 1 h at 4°C with agitation. Cell cycle and cell death analyses were performed on the MACSQuant flow cytometer (Miltenyi Biotec GmbH, Bergisch Gladbach, Germany).

### 2.7 Statistical Analysis

GraphPad Prism 9.5.1 was used for analysis (GraphPad Software Inc., San Diego, CA) unless otherwise indicated. Results are represented as mean ± standard error of the mean (SEM) of at least three independent experiments performed in triplicate. The Shapiro–Wilk test normality and an analysis of variance (ANOVA) with Dunnet's post‐test were donecarried out. The statistical significance of differences is indicated in figures by asterisks: a value of *p < 0.05 was considered statistically significant and a value of ****p < 0.0001 highly significant.

## 3 Results

### 3.1 Dose–Response Evaluation of Argentatins in Colorectal Cancer Cell Lines

**FIGURE 1:**
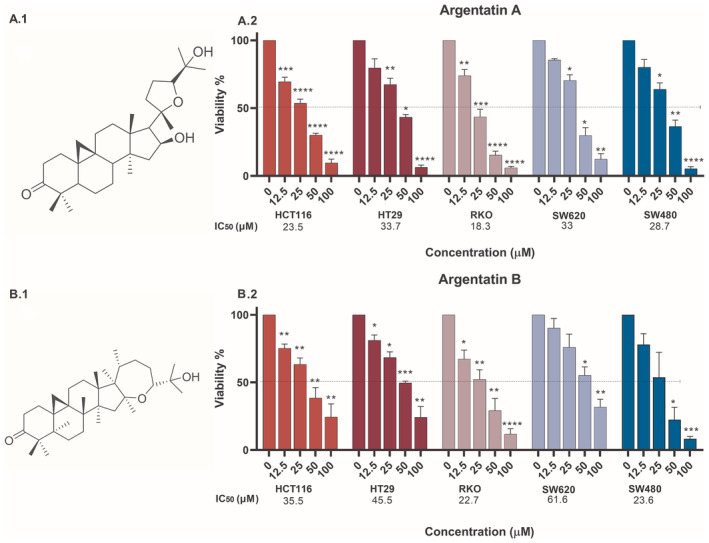
Cytotoxicity of argentatins A and B in a panel of colon cancer cell lines. Chemical structure of argentatins A (A.1) and B (B.1). Graphical representation of the mean percentage of MTT metabolised by cancer cells after treatment with different concentrations (μM) of argentatin A (A.2) and B (B.2) for 72 h, compared with the untreated control (DMSO), which was normalised to 100% cell viability. The mean of at least three independent experiments with their corresponding standard error of the mean (SEM) is shown. Significant differences with respect to the control are represented by an asterisk (*p < 0.05, **p < 0.005, ***p < 0.0005, ****p < 0.0001).

The cytotoxicity of argentatins has been reviously reported in the CRC cell lines HCT‐15, HCT‐116, RKO and SW‐620 [18, 20, 21]. In the present work, we have evaluated the cytotoxicity of increasing concentrations of argentatins A and B for 72 h using an MTT reduction assay in HT‐29 and SW‐480 lines, and in two HCT‐116 lines (wild type and resistant to 5‐fluorouracil). Single treatment with either compound resulted in significant cytotoxicity in all parental cell lines tested, with IC_50_ values ranging from 18.3 to 45.5 μM (Figure 1). As for the resistant cell model, these values were 34.4 and 36.3 μM for argentatins A and B, respectively (Figure S1).

### 3.2 Combination of Argentatins A and B Improves Cytotoxicity

**FIGURE 2:**
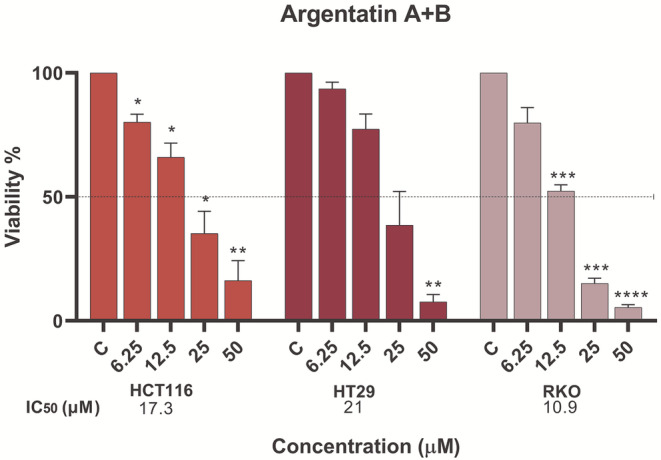
Cytotoxicity of argentatins A and B in combination in a panel of colon cancer cell lines. Graphical representation of the mean percentage of MTT metabolised by cancer cells after treatment with different concentrations (μM) for 72 h, compared with the untreated control (DMSO), which was normalised to 100% cell viability. The mean of at least three independent experiments with their corresponding standard error of the mean (SEM) is shown. Significant differences with respect to the control are represented by an asterisk (*p < 0.05, **p < 0.005, ***p < 0.0005, ****p < 0.0001).

Given the evident antiproliferative effects of argentatins A and B used as single agents, we next tested the cytotoxicity of the compounds in combination. We chose RKO, HT‐29 and HCT‐116 cells, which showed different degrees of inhibition in response to argentatins treatment, one with the highest, one with the lowest and one with intermediate inhibition, respectively, according to their IC_50_ values. The results showed that RKO cells remained the most susceptible to cytotoxicity induced by both compounds in combination (Figure 2), with an IC_50_ of 11.0 μM, which refers to each compound and not the sum of both. Notably, the efficacy of argentatin B was maintained, as its IC_50_ value when used alone was 22 μM, whereas only half of this concentration was required in combination with argentatin A. This may indicate that each compound retains its individual efficacy within the combined treatment. Similar results were observed for the other cell lines. Of note, both the intermediate response cell line (HCT‐116) and the lower inhibition cell line (HT‐29) also showed a better response, as evidenced by lower cell viability when argentatins A and B were combined.

### 3.3 Cell Viability in Response to 5‐Fluorouracil

**FIGURE 3:**
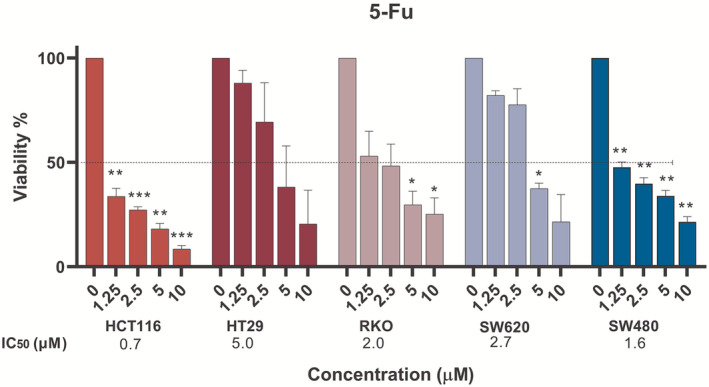
Cytotoxicity of 5‐fluorouracil in a panel of colon cancer cell lines. Graphical representation of the mean percentage of MTT metabolised by cancer cells after treatment with different concentrations (μM) of 5‐fluorouracil (5‐Fu) after 72 h compared with the untreated control (DMSO), which was normalised to 100% cell viability. The mean of at least three independent experiments with their corresponding standard error of the mean (SEM) is shown. Significant differences with respect to the control are represented by an asterisk (*p < 0.05, **p < 0.005, ***p < 0.0005).

We next sought to analyse the cellular response to argentatins A and B in combination with the classic mainstay of CRC chemotherapy, 5‐fluorouracil. We first tested the viability of the different cell lines in the presence of increasing concentrations of 5‐fluorouracil (1.25, 2.5, 5 and 10 μM) to determine its IC_50_ under the conditions tested for subsequent experiments. The cell line that showed the highest inhibition was HCT‐116 and the lowest inhibition was HT‐29, with IC_50_ values of 0.79 and 5.0 μM, respectively (Figure 3). On the other hand, treatment in the HCT‐116 (R) cell line showed a reduced cytotoxicity response, with and IC_50_ of 44.2 μM confirming its resistance profile to this drug (Figure S1).

### 3.4 Argentatins A and B in Combination With 5‐Fluorouracil Exert a Synergistic Effect

**FIGURE 4:**
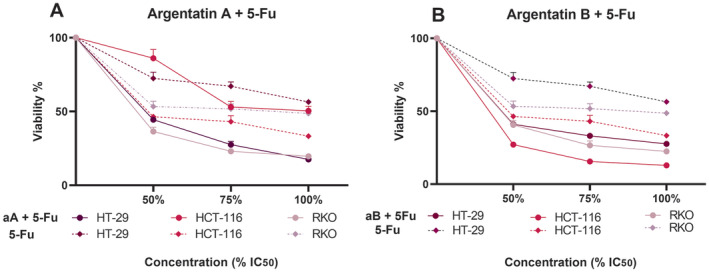
Cytotoxicity of 5‐fluorouracil in combination with argentatins in a panel of colon cancer cell lines. Graphical representation of the mean percentage of MTT metabolised by cancer cells after treatment with different concentrations (μM) of argentatin A plus 5‐fluorouracil (5‐Fu) (A) and argentatin B plus 5‐Fu (B) after 72 h compared with the corresponding untreated negative control (DMSO), which was normalised to 100% cell viability. The mean of at least three independent experiments with their corresponding standard error of the mean (SEM) is shown.

To test for synergy, we combined different concentrations of argentatins with 5‐fluorouracil, using as a reference the previously determined IC_50_ of each compound and then reducing their IC_50_ concentration to 75% and 50%. This approach indicated that the combined treatment of argentatin A or B with 5‐fluorouracil generally increased cytotoxicity in all cell lines tested (Figure 4), similar to the study combining argentatins A and B. The only exception was the null response observed in HCT‐116 cells when 5‐fluorouracil was combined with argentatin A, as cell viability was only reduced by 50% using the IC_50_ of both compounds, suggesting no improvement. Indeed, in the remaining cell lines, reducing the IC_50_ by one‐half, particularly in the argentatin A combinations, still led to substantial cytotoxicity (44% and 36% viability in HT‐29 and RKO cells, respectively) (Figure 4A). In contrast to the findings for the argentatin A combination, the combination of argentatin B and 5‐fluorouracil at the same concentrations reduced the viability of HCT‐116 cells to 27% (Figure 4B).

We used these three cell lines for the subsequent synergy assays to test whether the use of the compounds at a lower concentration resulted in a better response.

#### 3.4.1 Calculation of Synergy Distribution

The potential synergism/antagonism between argentatins and 5‐fluorouracil on RKO, HCT‐116 and HT‐29 cells was assessed with Combenefit software, using three different metrics of the several that were tested (Table S1), where SYN_MAX represents the maximum observed synergy and ANT_MAX represents the maximum observed antagonism. The SUM_SYN_ANT metric represents the sum of the net synergism and antagonism.

For the HCT‐116 cell line, argentatin A in combination with 5‐fluorouracil showed the greatest synergistic effect, with a metric of 31.30, followed by the RKO cell line with a metric of 30.93, which contrasts with the values of 41.18 and 6.97, respectively, obtained with the argentatin B and 5‐fluorouracil combination. As for the HT‐29 cell line, there were interestingly no significant differences with either of the two combinations (Figure S2B). However, the true synergistic effect was estimated by also considering the metrics related to the net antagonistic effect. Accordingly, net synergy was considered to occur when the difference between SYN_SUM and ANT_SUM (SUM_SYN_ANT) was greater than 1, indifferent in the range 0–1 and antagonistic when it was less than 0. Thus, argentatin A (SUM_SYN_ANT: +1.61), corresponding to the RKO cell line, and Argentatin B (SUM_SYN_ANT: +2.36), corresponding to HCT‐116 cell line, was considered to have a net synergistic effect when combined with 5‐fluorouracil (Figure S3).

To obtain the metrics for the program, we performed viability assays with a series of 9 concentration combinations for argentatin A or B plus 5‐fluorouracil. The percentage viability obtained was then processed by the Combenefit software to calculate the half maximal effective concentration (EC)_50_ of each compound, the values of which were very similar to the IC_50_ values calculated with GraphPad (Figure S3).

**FIGURE 5:**
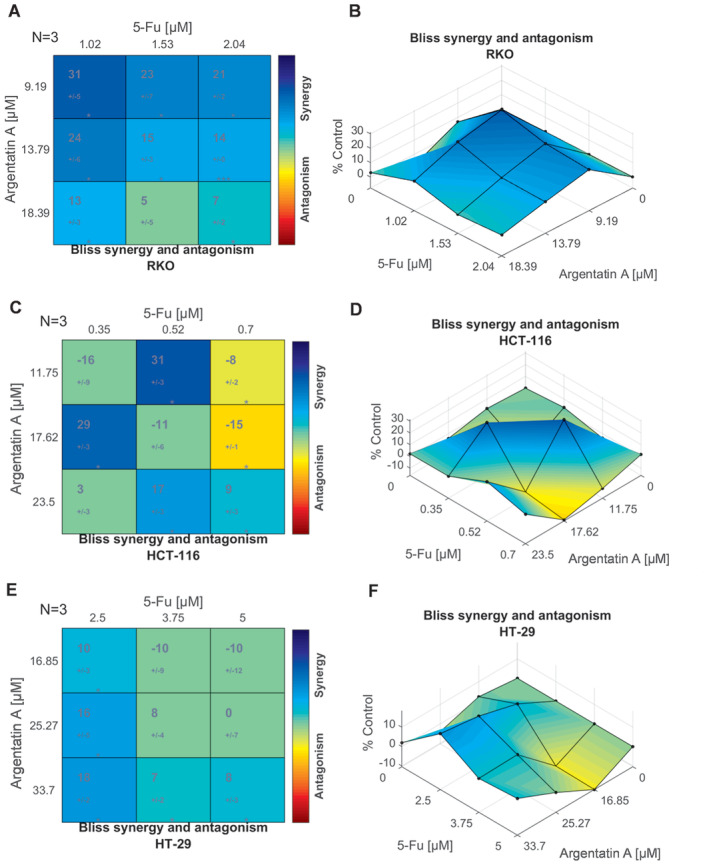
Interaction between argentatin A and 5‐fluorouracil. The synergy scores obtained using the Bliss Independence model are represented as a matrix (A, C and E), where the top values indicate the synergy score and the bottom value represents the associated standard deviation. The colour in each cell corresponds to the synergism (blue)/antagonism (red) scale. Green cell indicates non‐significance. The synergy scores of the combination of the nine concentrations are shown in a surface plot (B, D and F). Statistically significant results are represented by a *t*‐test of three independent experiments, *p < 0.05 and ***p < 0.001.

The synergism/antagonism score of each of the combinations of argentatin A and 5‐fluorouracil is shown in Figure 5. The results indicate that in the RKO cell line (Figure 5A), the combination of treatments showed significant areas of synergy, and the highest scores were obtained, especially using the lowest concentrations of both compounds, suggesting an improvement in the inhibition of cell viability compared to the individual treatments.

In the HCT‐116 cell line (Figure 5C), synergistic interactions were also observed, particularly in combinations of 11.75 with 0.52 μM and 17.62 with 0.035 μM of argentatin A and 5‐fluorouracil, respectively. However, for the HT‐29 cell line (Figure 5E), the combination of both agents showed the lowest scores, with synergy predominating at the lowest concentration of 5‐fluorouracil, regardless of the concentration of argentatin A utilised. The value of the scores is also detailed in Table S2.

The synergy scores were also plotted in a surface form (Figure 5B,D,F), with the peaks and valleys on the three‐dimensional surface representing the combinations of concentrations with the highest and lowest synergistic effect. In this representation, the behaviour of the combination of 5‐fluorouracil with argentatins was more evident, as shown by an elevation in the peak when using concentrations below the IC_50_ of both compounds, particularly when using one‐half of 5‐fluorourail concentrations.

**FIGURE 6:**
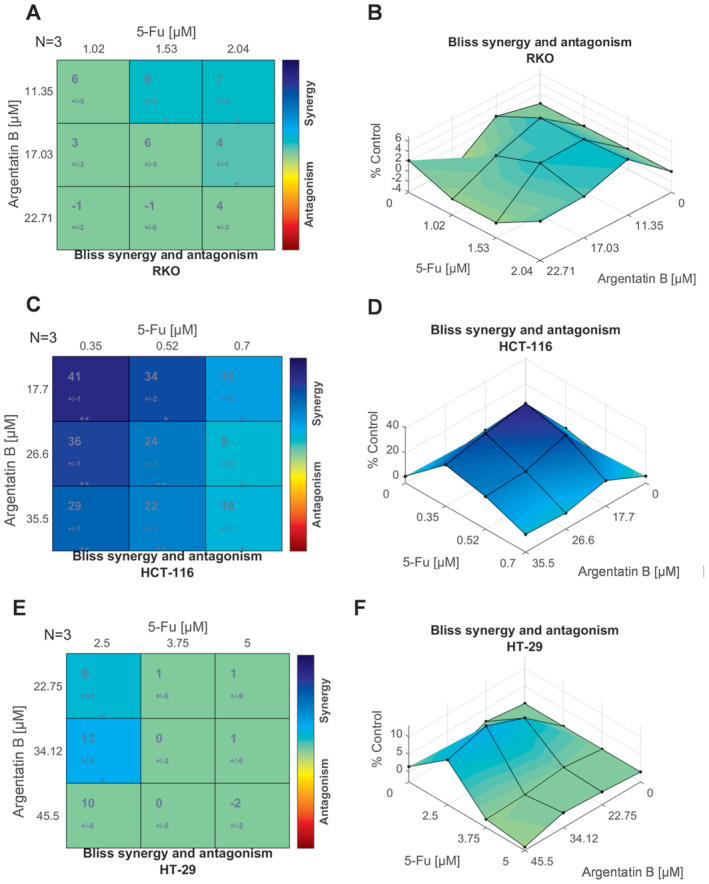
Interaction between argentatin B and 5‐fluorouracil. The synergy scores obtained using the Bliss Independence model are represented as a matrix (A, C and E), where the top values indicate the synergy score and the bottom value represent the associated standard deviation. The colour in each cell corresponds to the synergism (blue)/antagonism (red) scale. Green cell indicates non‐significance. The synergy scores of the combination of the nine concentrations are shown in a surface plot (B, C and F). Statistically significant results are represented by a t‐test of three independent experiments, *p < 0.05.

Concerning the combinations using argentatin B, for the RKO cell line, only 30% of the doses used show statistically significant differences (Figure 6A); a SYN_MAX value of +0.67 was obtained, with a synergistic effect of +0.39, so that in general, this combination does not suggest a notable synergistic effect. Notably, the lowest scores were obtained with the highest concentrations of each compound, which was also observed in the combinations with argentatin A. The mean synergism/antagonism scores can be found in Table S2. The same effect was observed in the HT‐29 cell line, because the value of the net synergistic effect corresponds to +0.32 (Figure S2A).

However, in the HCT‐116 cell line (Figure 6C), a considerable synergy between argentatin B and 5‐fluorouracil is observed in practically all the dose combinations used, reaching the highest scores with half of the IC_50_ of 5‐fluorouracil. This highlights its high sensitivity to this combination, and at the same time, a complementary mechanism of action or a potentiating effect of argentatin B together with 5‐fluorouracil, at least on this cell line.

The Bliss Independence model assumes a stochastic process in which the drugs do not interact, and the induced response is independent [25]. Although the combination argentatin B and 5‐fluorouracil was clearly superior in inhibiting cell viability (Figure 4B) compared with the 5‐fluorouracil alone, the mechanism of action appeared to be different with respect to argentatin A. As shown in Figure 6, in general, for argentatin B, there was no evident synergistic effect, except in the HCT‐116 cell line (at least using this model), as shown by the more uniform colour gradient and surface area (with no blue gradient). On the basis of the premise of the Bliss Independence model, it is possible that argentatin B has a similar mechanism of action to that of 5‐fluorouracil, or at least they act on the same pathway. There is also the possibility that this is due to an additive effect, and therefore, the synergism model is not appropriate, which is why the scores obtained were not sufficient to induce a peak in the plot.

For example, if both compounds inhibit the same enzyme or block the same cellular pathway, the combination would not necessarily increase the global effect because it is already saturated by the effect of one compound alone. This implies that the compounds do not significantly enhance each other, but neither do they significantly counteract each other. Accordingly, evaluating their interaction with other synergistic models could help to better understand their behaviour.

### 3.5 Cell Cycle Arrest by Argentatins and 5‐Fluorouracil Is Distinct

**FIGURE 7:**
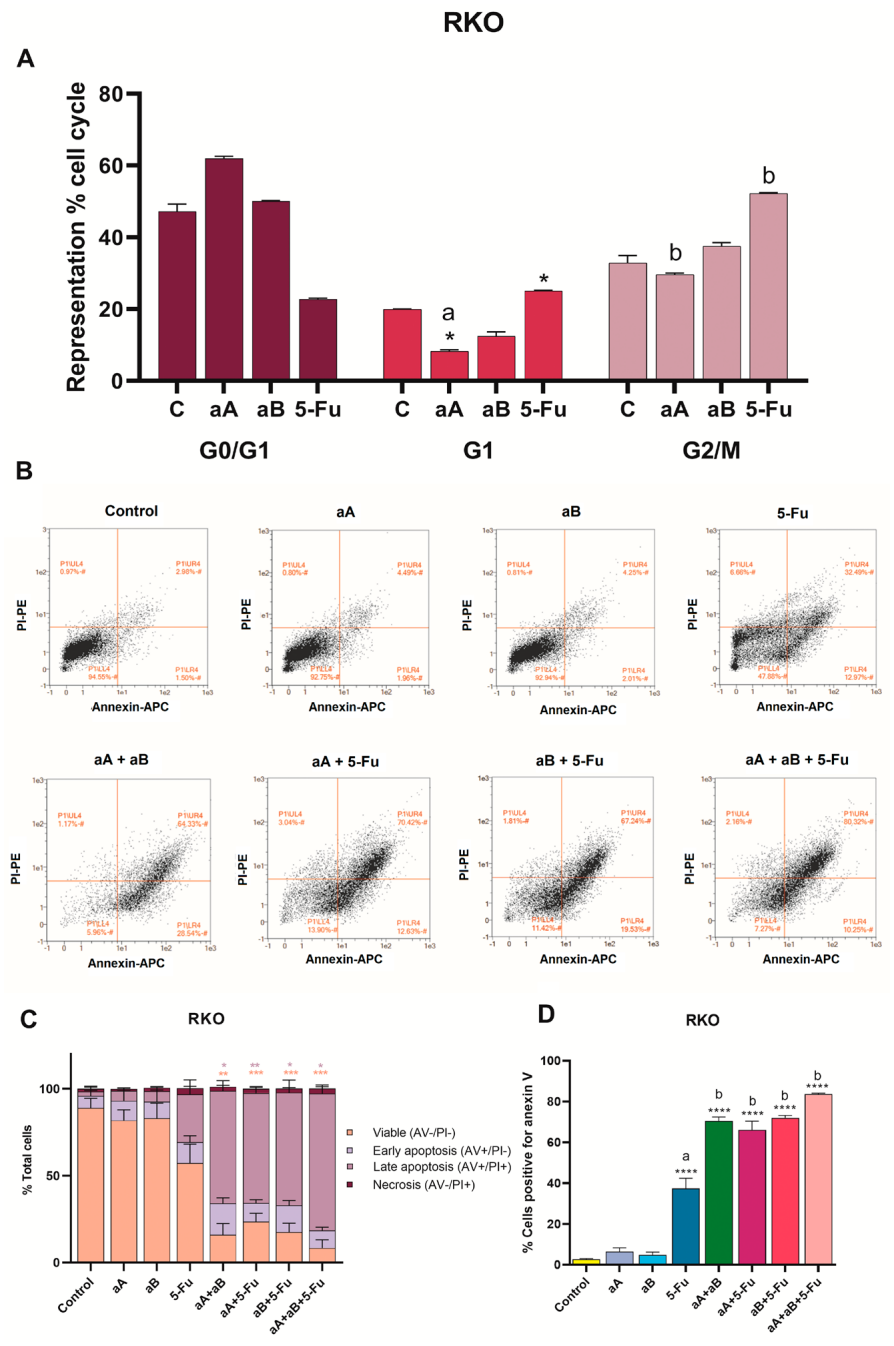
Cell death and cycle analysis in RKO cells. (A) Cell cycle analysis. Graphical representation of the percentage of cancer cells in each phase of the cell cycle after 24 h treatment with the IC_50_ of argentatin A, argentatin B and 5‐fluorouracil (5‐Fu). The mean of three independent assays is shown together with their respective standard error of the mean (SEM). The letters indicate the significant difference compared with that of the G0/G1 phase (a: *p* < 0.0001, b: *p* < 0.005). (B) Dot‐plot representation. The X and Y axes represent the fluorescence intensity of annexin (APC‐A) and propidium iodide (PI‐PE). The limiting zones are divided with each cell type according to labeling: P1/LL4 represents viable cells, P1/LR4 represents cells in early apoptosis, P1/UR4 represents cells in late apoptosis and P1/UL4 represent necrotic cells, after 24 h of treatment. (C) Apoptosis cell death assay. Percentage of live cells (AV‐/PI), cells in early apoptosis (AV+/PI), cells in late apoptosis (AV+/PI+) and necrotic cells (AV‐/PI+), versus the treatment with the IC_50_s of argentatin A, argentatin B, argentatin A + B, 5‐Fu, and their combinations. Asterisks indicate significant differences compared with the corresponding control, and the colours indicate the cell state (*p < 0.05, **p < 0.005, ***p < 0.0005). (D) Percentage of annexin V‐positive cells. Treatment of RKO cells with the IC_50_s of each compound and their combinations. (****p < 0.0001); the letters indicate the significant difference compared with the control (a) and with 5‐Fu (b).

To evaluate possible DNA damage and subsequent cycle arrest, we performed a cell cycle analysis 24 h after treatment using the IC_50_ of the compounds. Cells treated with argentatin A showed a significant cycle arrest of 62% in the G0/G1 phase, 14% greater than the control group, suggesting that the cells are quiescent. This contrasted with the results for 5‐fluorouracil, where a higher proportion of cells were in later phases, and only 22% were in G0/G1, almost half that of the control. Analysis of argentatin B treatment revealed no notable differences in any of the cell cycle phases when compared with that of the control group (Figure 7A).

The finding that 5‐fluorouracil treatment increases the proportion of cells in G2/M is consistent with its mechanism of action, which induces in DNA damage and activates cell cycle checkpoints, preventing cells from completing mitosis and arresting them in an attempt to repair the damage or to induce apoptosis if repair is not possible [26, 27, 28, 29].

The apparent cell cycle arrest induced by argentatin A in RKO cells prompted us to test whether it induced cell death by apoptosis. The compounds were tested both singly and in combination using the IC_50_ concentrations of each compound. Cells treated with argentatins A and B singly showed a similar proportion of viable cells with respect to the control, with no significant differences in the proportion of cells in early and late apoptosis (Figure 7B,C). By contrast, cells treated with 5‐fluorouracil alone showed a decrease in viability of ~30% compared with the control, which was accompanied by an increase in the number of cells in late apoptosis (Figure 7B,C).

The number of apoptotic cells increased considerably when the treatments were combined, as evidenced by the obvious stronger cell displacement towards the right quadrants of the scatter plots with the combination of argentatins A and B (Figure 7B). Likewise, the three double combinations showed a similar proportion of cells in late apoptosis, with a greater number of viable cells when argentatin A and 5‐fluorouracil were combined (Figure 7C).

We then evaluated the number of annexin V‐positive cells, corresponding to the number of cells undergoing apoptosis (Figure 7A). This confirmed that the combined treatments significantly increased the number of apoptotic cells, particularly the triple combination (~80%), which was 15% greater than the double treatments.

## 4 Discussion

Cancer treatment, particularly for CRC, is based on therapies that are often aggressive [30], and chemoresistance is a major challenge. The mainstay of CRC treatment, 5‐fluorouracil, has several side effects, including toxicity to both central nervous system progenitor cells and oligodendrocytes, even in the short term, limiting its long‐term clinical use [31, 32]. Accordingly, there is growing interest in the development of less‐intensive adjuvant treatments. In this regard, many studies are now investigating the clinical properties of compounds derived from natural sources such as plants, animals or microorganisms. These natural compounds have been used throughout history in the treatment of various diseases including certain cancers [33].

The triterpenes are biologically active compounds with proven in vitro antiproliferative activity against CRC cell lines [18]. Here, we investigated in vitro a potential treatment for CRC using triterpenes extracted from guayule, a plant native to northern México and southern USA [23].

Our analysis revealed a similar cytotoxicity of argentatin B in RKO cells as previously reported [20], with an IC_50_ of 22.7 μM versus 25.04 μM. We also found that argentatin A was more cytotoxic in the HCT‐116 and SW480 cell lines compared with that of a previous study [21], with an IC_50_ almost half of the reported 43.17 and 61.33 μM, respectively. In the HT‐29 and SW‐620 cell lines, the cytotoxic effect of argentatin A and argentatin B, respectively, was slightly lower. The differences between our results and those of previous works may be due to the different methodology used in each study to evaluate cell viability. In assays with HCT‐116 (R) cell line, there was a notable difference in response to treatment compared to the parental line. The IC_50_ of argentatin B in resistant cell line remained virtually the same as in the parental line, suggesting a preservation in its cytotoxic efficacy; however, the IC_50_ of argentatin A increased by ~45%.

Importantly, the combination of argentatin A and argentatin B exerted a greater cytotoxic effect than when used as single agents. Of the two triterpenes, the most significant change was found with argentatin B. For example, the IC_50_ of argentatin B in HT‐29 and RKO cells decreased from 45.5 μM to 21 μM and from 22.7 to 10.9 μM, respectively, when combined with argentatin A, a decrease of ~50%. For argentatin A, we noted decreases in the IC_50_ of 25% to 40% compared with its single use.

We also observed superior cytotoxicity when the argentatins were combined with 5‐fluorouracil, as a 50% decrease in the IC_50_ concentration of both compounds resulted in lower cell viability compared with their single use. In the three cell lines in which the combination of drugs (5‐fluorouracil with each of the argentatins) was tested, the concentration of 5‐fluorouracil used ranged from 5 to 0.39 μM, depending on the IC_50_ of each cell line, with the latter concentration used in HCT‐116 cells. This is substantially lower than what has been used in other studies in which synergistic effects have been demonstrated when using 5‐fluorouracil with other compounds, such as diosmetin and kaempferol [34, 35].

It should be noted that some of the drugs with which 5‐fluorouracil was combined were used in very high concentrations compared with our study, for example, 100 μM for kaempferol and 50 μM for tenacissocide G [36, 37]. We found that the concentration of argentatins that exerted a synergistic effect ranged from 9.19 μM for argentatin A to 17.76 μM for argentatin B. This is an important finding and highlights the potential of 5‐fluorouracil combinations with argentatins to be used at relatively low concentrations, which may translate into lower systemic toxicity and less side effects in a therapeutic context.

The pronounced difference in the synergistic effects using the Combenefit software when combining argentatins with 5‐fluorouracil could be due to several factors related to the mechanisms of action of each compound and their interaction with 5‐fluorouracil. The Bliss Independence model predicts the combination of effects based on the probability that both compounds act separately and that their effects are independent. If both compounds have similar mechanisms of action or affect the same cellular pathway, the model may not detect true synergy, although a reduction in cell viability is observed [38]. Argentatin A may be acting on a different or complementary pathway to that of 5‐fluorouracil, which results in a significant synergistic effect.

On the other hand, the results concerning the combination with argentatin B on the HCT‐116 cell line highlight that the synergistic response is highly dependent on the intrinsic characteristics of the cell line used, the underlying molecular mechanisms and the genetic context. A particular feature of this cell line is a deficiency in the DNA mismatch repair (MMR) system, which generates microsatellite instability and a higher level of accumulated mutations. As a result, it could be responding more sensitively to the cumulative damage caused by 5‐fluorouracil, especially [38, 39]. In contrast to the RKO and HT‐29 cell lines, the combination of argentatin B and 5‐fluorouracil could be additive rather than synergistic, and treatment with both compounds produces an effect that is equal to the sum of the individual effects. Although this may result in increased cytotoxicity in cell viability assays, it does not necessarily indicate synergy according to the strict criteria of the Bliss model [36, 40]. Thus, further studies exploring the variables described above are needed to better understand the differences between the argentatins.

Another possibility involves the intracellular distribution of argentatins, affecting the availability and efficacy of each compound in combination with 5‐fluorouracil; in this sense, argentatin A may have better penetration or stability.

The use of natural compounds in enhancing chemotherapy has the potential to prevent resistance by affecting more than one target. Indeed, the pathogenesis of CRC involves various cell cycle alterations that promote its establishment and development, in addition to inflammation, abnormal DNA methylation, microRNAs triggers or chromosomal instability characterised as widespread loss of heterozygosity [41].

We found that argentatin A treatment affected the cell cycle of CRC cells, inducing a quiescent or resting state. This is justified by the fact that there is a marked increase in the cell population in the G0/G1 phase, almost 15% more, compared to that of the control. The finding that the proportion of cells in G1 was lower in argentatin A‐treated cells than that in control cells is counter intuitive; however, this likely indicates that cells arrest mainly in the G0 phase. In contrast to argentatin A, no significant effect on the cell cycle was found after argentatin B treatment.

The effect of argentatin A on the cell cycle might be counterproductive in a clinical setting, as this phase is generally reversible. Cancer cells that enter G0 can become quiescent or senescent. If cells choose quiescence, they can become more resistant to chemotherapy, which typically targets cells that are actively dividing. Furthermore, if they choose a dormant state, this can lead to recurrence after a period of remission. However, if cells opt for senescence, they cannot return to the cell cycle [42].

Immunocytometry analysis confirmed that all the tested combinations induced significant apoptosis in RKO cells, especially the triple combination, and argentatin co‐treatment substantially enhanced the apoptotic effect of 5‐fluorouracil (~60% apoptosis), as 5‐fluorouracil alone induces apoptosis by approximately 25% compared to the control group. This increase in apoptosis was similar to that seen when using the combination of argentatins A and B, suggesting that they are effective in inducing apoptosis. These data appear to be superior to those of other studies [34, 35, 37, 43], in which the combination of 5‐fluorouracil with diosmetin induced 45% apoptosis [35], and very similar to those reported when combined with allicin [In the present work, we have evaluated the cytotoxicity of increasing concentrations of argentatins A and B for 72 h using an MTT reduction assay in HT‐29 and SW‐480 lines, and in two HCT‐116 lines (wild type and resistant to 5‐fluorouracil) [44].], but at substantially higher concentrations than ours, 50‐fold higher for 5‐fluorouracil and 2‐fold higher for allicin.

Although not significant, we noted that 5‐fluorouracil increased the proportion of necrotic cells two‐fold over the control. In a clinical context, this increase could lead to an inflammatory response and additional tissue damage, which contrasts with the less inflammatory (and more controlled) apoptosis [38, 39, 40]. Thus, the combination of the argentatins has the advantage of activating the apoptosis pathway, with less impact on necrosis. Indeed, apoptosis plays a key role in correcting normal tissue stability, and thus, therapy options targeting apoptosis may be more effective in preventing CRC progression.

Overall, our findings provide potentially meaningful clinical guidelines for the application of drug combinations in cancer treatments. Furthermore, they offer valuable information on possible interconnected mechanisms of cell responses to argentatins. However, further studies are needed to deepen our understanding of these mechanisms and to optimise therapeutic strategies based on these drug combinations.

## 5 Conclusions

We investigated the use of argentatins as a potential treatment for CRC. In addition to their antiproliferative effects, these compounds reduced the need for high doses of chemotherapy due to their synergistic effects with 5‐fluorouracil, which has the potential to minimise the side effects often associated with conventional chemotherapy. Our results support that both argentatins A and B can reduce cell viability and, when combined in vitro, also induce apoptosis. Argentatin A appears to induce quiescence, with significant cell cycle arrest at G0/G1. When argentatins were combined with 5‐fluorouracil, we found a greater proportion of cells in late apoptosis than when the antimetabolite was used alone. Future studies should elucidate the mechanism of action of argentatins, which may open up promising avenues for the development of more effective therapies for CRC and expand the arsenal of treatments available to combat this disease.

## Author Contributions

**Paula Sánchez‐Olivares:** investigation (equal), writing – original draft (equal). **Aniela M. Silva‐Nolasco:** investigation (equal), writing – original draft (equal). **Miguel A. de la Cruz‐Morcillo:** conceptualization (equal), data curation (equal), methodology (equal). **María Mercedes García‐Martínez:** formal analysis (equal), investigation (equal). **Alejandro Pinedo‐Serrano:** investigation (equal). **Manuel Carmona:** conceptualization (equal), project administration (lead), supervision (equal), writing – review and editing (equal). **Eva M. Galán‐Moya:** conceptualization (equal), supervision (equal), writing – review and editing (equal).

## Conflicts of Interest

The authors declare no conflicts of interest.

## Supporting information


**Figure S1.** Cytotoxicity of argentatins and 5‐fluorouracil in a colon cell line resistant to 5‐fluorouracil. Graphical representation of the mean percentage of MTT metabolised by cancer cells after treatment with different concentrations (μM) of argentatins and 5‐fluorouracil for 72 h, compared with the untreated control (DMSO), which was normalised to 100% cell viability. The mean of at least three independent experiments with their corresponding standard error of the mean (SEM) is shown. Significant differences with respect to the control are represented by an asterisk (*p < 0.05, **p < 0.005, ***p < 0.0005).

**Figure S2.** Graphical representation of three of the metrics generated by the Combenefit software for the Bliss independence model using the combination of argentatin A or B with 5‐fluorouracil in a panel of colon cancer cells. SYN_MAX is the maximum observed synergy, and the ANT_MAX is the maximum observed antagonism (A). SUM_SYN_ANT is the sum of the net synergism and antagonism (B) and represents all values within the dose space.

**Figure S3.** Dose‐–response curve plot of argentatins and 5‐fluorouracil in a panel of colon cancer cell.

**Table S1.** Metrics obtained by the Combenefit 2.021 program for the combination of argentatin A with 5‐Fu and argentatin B with 5‐fluorouracil in a panel of colon cancer cells by the Bliss method.

**Table S2.** Mean synergism/antagonism obtained by the Combenefit 2.021 program for the combination of argentatin A with 5‐fluorouracil (5‐Fu) and argentatin B with 5‐Fu in a panel of colon cancer cells by the Bliss method.

## Data Availability

The data that support the findings of this study are available from the corresponding author upon reasonable request.
