# Supplementary figures and images for: Synergistic Potential of Argentatins A and B to Improve 5‐Fluorouracil Cytotoxicity in Colorectal Cancer Cell Models
Source: J Cell Mol Med. 2024 Dec 20;28(24):e70294. doi: 10.1111/jcmm.70294 (PMC11662136; doi:10.1111/jcmm.70294)

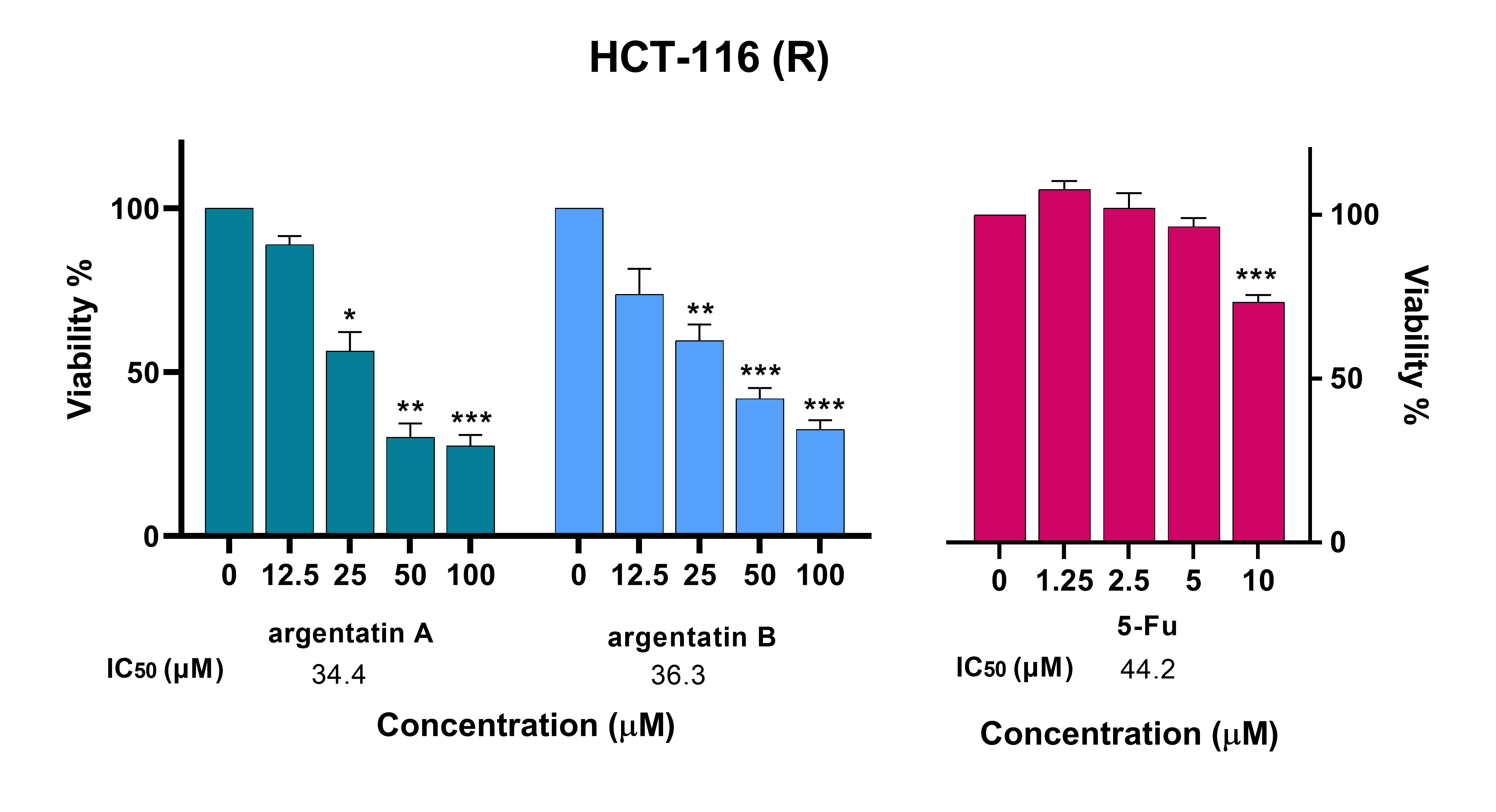

Supplement: Supplementary file 1 — Figure S1. Cytotoxicity of argentatins and 5‐fluorouracil in a colon cell line resistant to 5‐fluorouracil. Graphical representation of the mean percentage of MTT metabolised by cancer cells after treatment with different concentrations (μM) of argentatins and 5‐fluorouracil for 72 h, compared with the untreated control (DMSO), which was normalised to 100% cell viability. The mean of at least three independent experiments with their corresponding standard error of the mean (SEM) is shown. Significant differences with respect to the control are represented by an asterisk (*p < 0.05, **p < 0.005, ***p < 0.0005). [file JCMM-28-e70294-s001.tif]

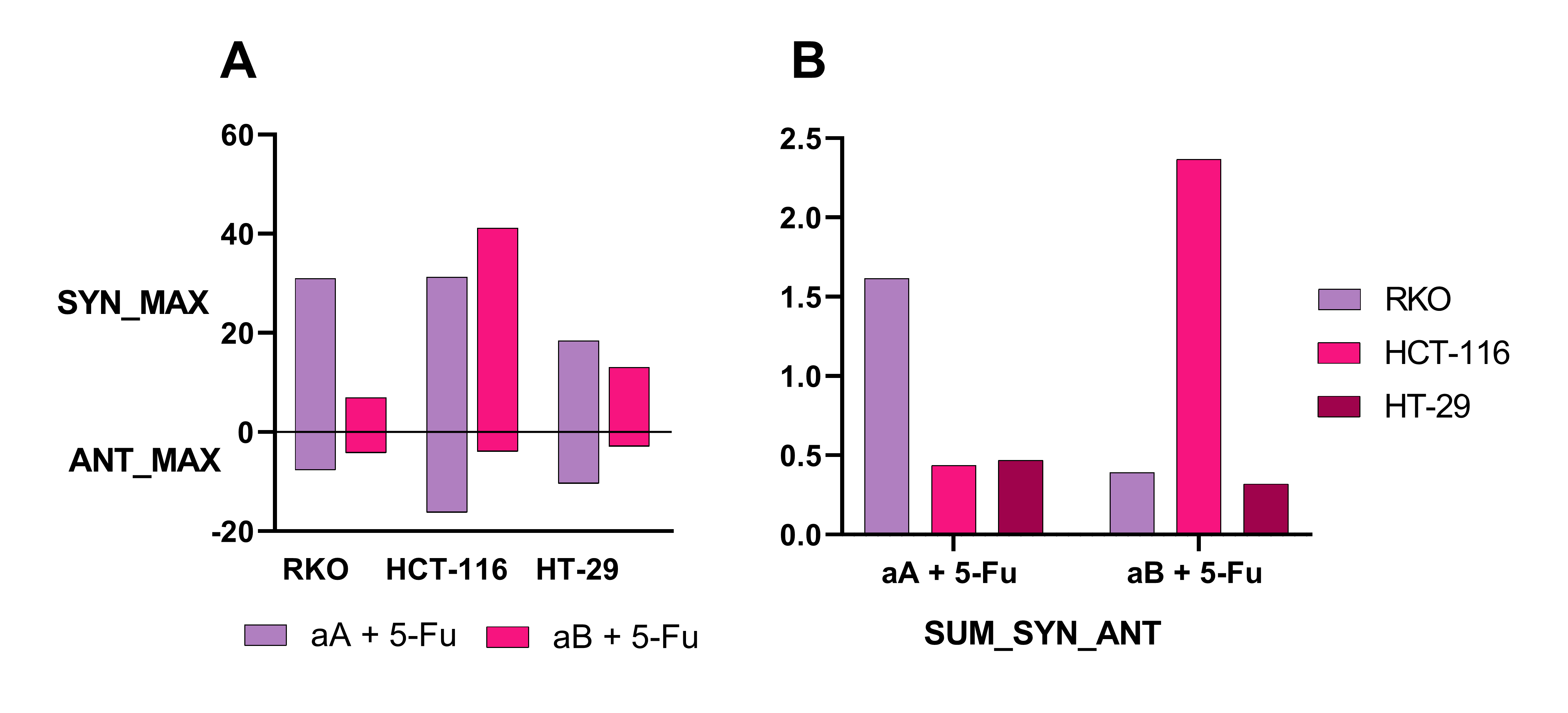

Supplement: Supplementary file 2 — Figure S2. Graphical representation of three of the metrics generated by the Combenefit software for the Bliss independence model using the combination of argentatin A or B with 5‐fluorouracil in a panel of colon cancer cells. SYN_MAX is the maximum observed synergy, and the ANT_MAX is the maximum observed antagonism (A). SUM_SYN_ANT is the sum of the net synergism and antagonism (B) and represents all values within the dose space. [file JCMM-28-e70294-s002.tif]

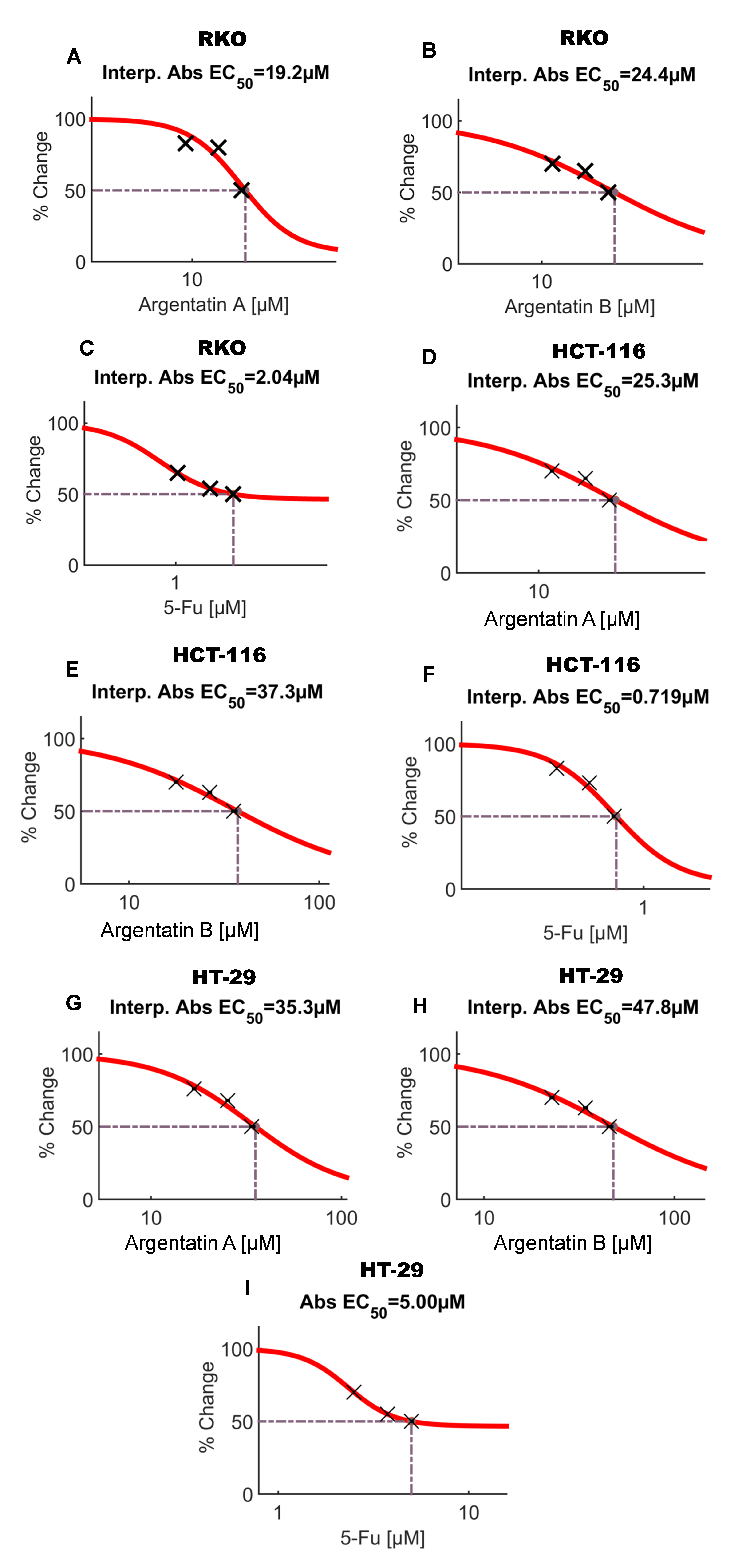

Supplement: Supplementary file 3 — Figure S3. Dose‐–response curve plot of argentatins and 5‐fluorouracil in a panel of colon cancer cell. [file JCMM-28-e70294-s003.tif]
