# Supplementary material for: Synergistic Potential of Argentatins A and B to Improve 5‐Fluorouracil Cytotoxicity in Colorectal Cancer Cell Models
Source: J Cell Mol Med. 2024 Dec 20;28(24):e70294. doi: 10.1111/jcmm.70294 (PMC11662136; doi:10.1111/jcmm.70294)
Supplement: Supplementary file 4 — Table S1. Metrics obtained by the Combenefit 2.021 program for the combination of argentatin A with 5‐Fu and argentatin B with 5‐fluorouracil in a panel of colon cancer cells by the Bliss method. [file JCMM-28-e70294-s005.docx]

|  | **RKO** | | **HCT-116** | | **HT-29** | |
| --- | --- | --- | --- | --- | --- | --- |
| **Metrics** | **aA/5-Fu** | **aB/5-Fu** | **aA/5-Fu** | **aB/5-Fu** | **aA/5-Fu** | **aB/5-Fu** |
| SYN MAX | 30.9374431 | 6.9734042 | 31.3022248 | 41.1883429 | 18.4311831 | 13.1165902 |
| SYN SUM | 1.6148037 | 0.4083582 | 1.000016778 | 2.3675314 | 0.6622387 | 0.3321703 |
| SYN SUM WEIGHTED | 1.1181621 | 0.2630644 | 0.494869823 | 1.9476255 | 0.4209876 | 0.2121053 |
| SYN SPREAD | 0.2284639 | 0.2419904 | 0.178737649 | 0.2397511 | 0.1895530 | 0.1591365 |
| SYN_AVERAGE_C1 | 11.9810856 | 14.4998635 | 15.83395383 | 24.0886211 | 26.2695353 | 32.0431627 |
| SYN_AVERAGE_C2 | 1.3707014 | 1.4920171 | 0.450924577 | 0.4573400 | 2.9962672 | 0.3713623 |
| ANT MAX | -7.6470924 | -4.1418104 | -16.17695237 | -3.8680300 | -10.3942250 | -2.8240927 |
| ANT SUM | 0 | -0.0161759 | 0.563505413 | 0 | -0.1919085 | -0.0115504 |
| ANT_SPREAD | 0 | 0.0624942 | 0.186638267 | 0 | 0.1358786 | 0.0639528 |
| ANT_AVERAGE_C1 | 0 | 22.71 | 15.62224959 | 0 | 16.9145935 | 45.5 |
| ANT_AVERAGE_C2 | 0 | 1.3405465 | 0.528444378 | 0 | 4.0964917 | 4.4904281 |
| ANT SUM WEIGHTED | 0 | -0.0054450 | 0.265662569 | 0 | -0.0949749 | -0.0037186 |
| SUM SYN ANT | 1.6148037 | 0.3921822 | 0.436511364 | 2.3675314 | 0.4703301 | 0.3206198 |
| SUM SYN ANT WEIGHTED | 1.1181621 | 0.2576193 | 0.229207254 | 1.9476255 | 0.3260127 | 0.2083866 |
