# Supplementary material for: Synergistic Potential of Argentatins A and B to Improve 5‐Fluorouracil Cytotoxicity in Colorectal Cancer Cell Models
Source: J Cell Mol Med. 2024 Dec 20;28(24):e70294. doi: 10.1111/jcmm.70294 (PMC11662136; doi:10.1111/jcmm.70294)
Supplement: Supplementary file 5 — Table S2. Mean synergism/antagonism obtained by the Combenefit 2.021 program for the combination of argentatin A with 5‐fluorouracil (5‐Fu) and argentatin B with 5‐Fu in a panel of colon cancer cells by the Bliss method. [file JCMM-28-e70294-s004.docx]

|  |  | **RKO** | | |
| --- | --- | --- | --- | --- |
|  |  | **5-Fu** | | |
|  |  | **1.0 µM** | **1.5 µM** | **2.0 µM** |
| **aA** | **9.1 µM** | 30.937443 | 23.329962 | 20.857535 |
|  | **13.7 µM** | 24.291651 | 15.303708 | 14.196984 |
|  | **18.3 µM** | 12.892203 | 5.3094909 | 6.8744905 |

|  |  | **RKO** | | |
| --- | --- | --- | --- | --- |
|  |  | **5-Fu** | | |
|  |  | **1.0 µM** | **1.5 µM** | **2.0 µM** |
| **aB** | **11.3 µM** | 6.2044218 | 6.4474511 | 6.9734042 |
|  | **17.0 µM** | 3.1622377 | 6.2434224 | 4.0986423 |
|  | **22.7 µM** | -0.95834205 | -1.1589062 | 3.665235 |

|  |  | **HT-29** | | |
| --- | --- | --- | --- | --- |
|  |  | **5-Fu** | | |
|  |  | **0.3 µM** | **0.5 µM** | **0.7 µM** |
| **aA** | **16.8 µM** | 10.277859 | -10.037567 | -10.394225 |
|  | **25.2 µM** | 15.766039 | 7.606514 | -0.19269231 |
|  | **33.7 µM** | 18.431183 | 7.093747 | 8.4903184 |

|  |  | **HT-29** | | |
| --- | --- | --- | --- | --- |
|  |  | **5-Fu** | | |
|  |  | **0.3 µM** | **0.5 µM** | **0.7 µM** |
| **aB** | **22.7 µM** | 8.8880366 | 1.2886491 | 0.80527732 |
|  | **34.1 µM** | 13.116590 | 0.010082291 | 1.3777349 |
|  | **45.5 µM** | 9.9564483 | -0.45876023 | -1.8529638 |

|  |  | **HCT-116** | | |
| --- | --- | --- | --- | --- |
|  |  | **5-Fu** | | |
|  |  | **0.3 µM** | **0.5 µM** | **0.7 µM** |
| **aA** | **11.75 µM** | -16.176952 | 31.302225 | -7.9240322 |
|  | **17.62 µM** | 28.855363 | -10.931240 | -15.285120 |
|  | **23.5 µM** | 2.5347148 | 17.271768 | 8.9012757 |

|  |  | **HCT-116** | | |
| --- | --- | --- | --- | --- |
|  |  | **5-Fu** | | |
|  |  | **0.3 µM** | **0.5 µM** | **0.7 µM** |
| **aB** | **17.75 µM** | 41.188343 | 34.332075 | 16.375495 |
|  | **26.62 µM** | 35.770340 | 24.045419 | 8.8613753 |
|  | **35.5 µM** | 28.942491 | 22.103895 | 10.174837 |
